# Exposure to Antineoplastic Drugs in Occupational Settings: A Systematic Review of Biological Monitoring Data

**DOI:** 10.3390/ijerph19063737

**Published:** 2022-03-21

**Authors:** Veruscka Leso, Cristina Sottani, Carolina Santocono, Francesco Russo, Elena Grignani, Ivo Iavicoli

**Affiliations:** 1 Department of Public Health, Section of Occupational Medicine, University of Naples Federico II, Via S. Pansini 5, 80131 Naples, Italy; veruscka.leso@unina.it (V.L.); carolinasantocono4@gmail.com (C.S.); francesco.russo.na@gmail.com (F.R.); 2 Environmental Research Center, Istituti Clinici Scientifici Maugeri IRCCS, Via Salvatore Maugeri, 10, 27100 Pavia, Italy; cristina.sottani@icsmaugeri.it (C.S.); elena.grignani@icsmaugeri.it (E.G.)

**Keywords:** cytotoxic drugs, antiblastic drugs, job exposure, healthcare workers, exposure evaluation, biomarkers, internal dose, human biomonitoring, risk assessment, risk management

## Abstract

The high toxicity of antineoplastic drugs (ADs) makes them dangerous not only for patients, but also for exposed workers. Therefore, the aim of this review was to provide an updated overview of the biological monitoring of occupational AD exposure in order to extrapolate information useful to improve risk assessment and management strategies in workplaces. Several studies demonstrated that remarkable portions of healthcare workers may have traces of these substances or their metabolites in biological fluids, although with some conflicting results. Nurses, directly engaged in AD handling, were the occupational category at higher risk of contamination, although, in some cases, personnel not involved in AD-related tasks also showed quantifiable internal doses. Overall, further research carried out on greater sample sizes appears necessary to gain deeper insight into the variability retrieved in the reported results. This may be important to understand the impact of the extent of ADs use, different handling, procedures, and cleaning practices, spill occurrence, training of the workforce, as well as the adoption of adequate collective and personal protective equipment in affecting the occupational exposure levels. This may support the achievement of the greatest clinical efficiency of such therapies while assuring the health and safety of involved workers.

## 1. Introduction

Antineoplastic drugs (ADs), also known as chemotherapy or cytotoxic drugs, include compounds with various mechanisms of action that are used to fight the global burden of cancer, preventing or disrupting cell division of neoplastic cells [1]. However, their action on malignant cells is only partially selective and normal ones may also be affected, leading to significant toxic side effects [2]. For more than three decades, researchers have documented AD toxicity [3,4] and, in 2004, the National Institute for Occupational Safety and Health (NIOSH) issued an alert summarizing their health effects, including skin rashes, adverse reproductive outcomes, hematopoietic and other cancers [5]. In fact, most ADs are classified as carcinogenic (group 1) by the International Agency for Research on Cancer (IARC) and many of them have been recognized as probably (group 2A) or possibly carcinogenic (group 2B) for humans [6].

Their high toxicity makes ADs dangerous not only for patients, but also for all the workers that are, to different extents, involved in their handling. Workplace exposure may occur in manufacturing, distribution, receipt, storage, transport, compounding, and administration, as well as during waste handling and care of treated patients [7]. Therefore, pharmacists and pharmacy assistants, nurses, physicians, environmental service workers (e.g., janitors and caretakers), shippers and receivers, industrial laundry workers, and pharmaceutical manufacturing workers can all be exposed to such dangerous drugs [8]. These workers may be exposed primarily through dermal contact, but also via ingestion, inhalation, and accidental injection, to small doses of a broad range of cytotoxic drugs over decades, in some cases every workday, year after year [9].

This has inevitably raised occupational health concerns considering the high total number of licensed anticancer drugs (270), of which 90% (243) were approved by the US Food and Drug Administration (FDA), 62% (168) by the European Medicines Agency and 19% (50) by different European national approvals; over 18 million chemotherapy doses are administered annually in the US alone [10]. Moreover, an increasing number of preparations and administrations of cytotoxic drugs has been reported worldwide, and an increasing variety of healthcare workers are expected to be potentially exposed due to the rapidly expanding use of these agents in non-oncology practices for treating non-malignant diseases [8].

For all these reasons, several efforts have been made to reduce or eliminate ADs environmental contamination and, consequently, occupational exposure, through advanced engineering support such as robotic systems, closed system drug transfer devices, and compounding aseptic containment isolators [11,12,13,14,15,16,17], and by improving safe drug handling practices and personal protective equipment (PPE) among workers [5,18,19]. Nevertheless, it is evident that the potential exposure to ADs cannot be completely eliminated. In the last four decades, several studies have reported detectable and/or quantifiable concentrations of such hazardous drugs both in workplaces and in biological matrices of engaged employees despite the development of suitable strategies to assess risks for healthcare workers and the adoption of preventive and protective measures [20,21,22,23,24]. However, the current lack of globally harmonized standards for the prevention of AD exposure makes such an ever-worrisome problem far from being solved and a still present occupational health priority [13,25].

Therefore, the primary aim of our review was to provide an updated overview of currently available data on the occupational AD exposure assessed through the biological monitoring of engaged workers. This will provide the opportunity to assess the effectiveness of the currently adopted measures to control exposure and collect data that may be helpful for their improvement. Additionally, secondary purposes will be focused on identifying those tasks and job procedures at increased risk of exposure in order to define updated as well as tailored risk assessment and management procedures in occupational settings, including the implementation of biological monitoring programs, and to increase awareness in the workforce, in order to specifically assure the safety and health of involved workers.

## 2. Materials and Methods

The systematic literature search was performed following the Preferred Reporting Items for Systematic Reviews and Meta-Analyses Statement (PRISMA) criteria [26] (Supplementary Materials Figure S1). Research articles, published in English language and exploring AD exposure through biological monitoring in occupational settings were considered suitable for review. Therefore, we considered as eligible studies those enrolling workers involved in AD handling, along all the possible drug use processes (i.e., manufacture, receipt, transport, preparation, administration, cleaning, laundering, waste management, etc.). We excluded reviews, notes, book chapters, letters, editorials, conference papers, as well as articles published in languages other than English and, more generally, any study that did not provide biological monitoring data on exposure to ADs in workers.

PubMed, Scopus, ISI Web of Knowledge, as principal databases, and forward and backward citations were searched for studies published between 1 January 2015 and 31 December 2021. We developed database-specific search strategies including a combination of keywords. The following key search terms were used in strategies specific to each database: “Antineoplastic drug*” “Chemotherap*” OR “Antiblastic drug*” OR “Hazardous drug*” combined through the Boolean operator “AND” with the terms “occupational exposure”.

The first step of the search strategy, consisting of identifying the articles of interest for review, retrieved 200, 183 and 235 records on PubMed, Scopus, and ISI Web of Science databases, respectively. After removal of duplicates, 2 researchers, C.S., F.R., independently reviewed titles and abstracts of all identified articles (318) and discussed inconsistencies until a consensus was obtained. A total of 298 articles were excluded as they were off topic for title and abstract analysis (266), because they were review articles, letters to the Editor, conference abstracts and book chapters (30), or because they were published in languages other than English (2). Then, the full text of the remaining 20 articles were screened for inclusion by these two researchers independently. In case of disagreement, in this phase, consensus on inclusion and exclusion was reached by discussion and, if necessary, a third researcher, I.I., was consulted. The citation pool of relevant publications identified in the literature search was further enlarged by assessing the reference list accompanying the selected articles; this allowed the inclusion of 6 additional eligible papers. Overall, our search retrieved a total of 26 articles suitable for review.

Key information about the included studies was collected in a standardized data extraction form independently by three of the authors, C.S., F.R. and V.L., and extracted data were then compared in order to exclude any possible inaccuracy during the process. Relevant issues analyzed included: the study population (workplace setting; occupational categories explored, size of the target population, control groups when available); ADs under investigation; studied biomarkers and biological matrices explored; sampling strategy (timing of samples collection, i.e., pre- and post-shift sampling, spot sampling); applied analytical methods (techniques, limit of detection (LOD), limit of quantification (LOQ)); results obtained in terms of percentages of positive samples retrieved among the investigated population; portions of positive workers or ADs concentration in biological matrices, when available.

Three of the authors, C.S., F.R. and V.L., independently evaluated the quality of the selected studies using the Newcastle–Ottawa quality assessment scale [27]. When these three authors disagreed on the evaluation, the remaining authors also reviewed the article, and the judgement made by the majority of the reviewers determined the quality rating. Based on a maximum of nine points attributable within three different sections (Selection, Comparability and Outcome), a range scale was adopted, going from a sufficient evaluation with 6 points, a good evaluation for 7–8 points and an excellent evaluation for 9 points; the final evaluation was decided via discussion (Table 1).

## 3. Results

**Figure 1:**
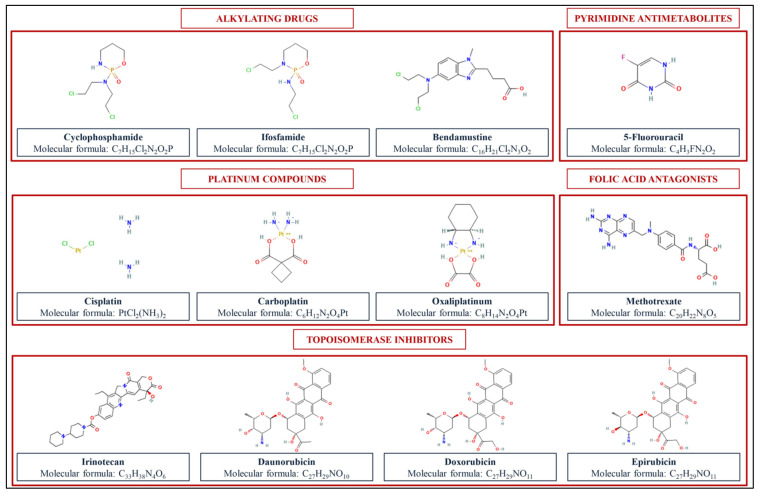
Chemical structures of the main ADs investigated by the studies object of this systematic review. The 2D structure images of the ADs in this figure were obtained from PubChem as follows: Cyclophosphamide: PubChem Identifier CID 2907 (https://pubchem.ncbi.nlm.nih.gov/compound/2907#section=2D-Structure (accessed on 17 March 2022); Iphosphamide: PubChem Identifier CID 3690 (https://pubchem.ncbi.nlm.nih.gov/compound/3690#section=2D-Structure (accessed on 17 March 2022)); Bendamustine: PubChem Identifier CID 65,628 (https://pubchem.ncbi.nlm.nih.gov/compound/65628#section=2D-Structure (accessed on 17 March 2022)); 5-Fluorouracil: PubChem Identifier CID 3385 (https://pubchem.ncbi.nlm.nih.gov/compound/3385#section=2D-Structure (accessed on 17 March 2022)); Cisplatin: PubChem Identifier CID 5,702,198 (https://pubchem.ncbi.nlm.nih.gov/compound/5702198#section=2D-Structure (accessed on 17 March 2022)); Carboplatin: PubChem Identifier CID 426,756 (https://pubchem.ncbi.nlm.nih.gov/compound/426756#section=2D-Structure (accessed on 17 March 2022)); Oxaliplatinum: PubChem Identifier CID 43,805 (https://pubchem.ncbi.nlm.nih.gov/substance/135005397#section=2D-Structure (accessed on 17 March 2022)); Methotrexate: PubChem Identifier CID 126,941 (https://pubchem.ncbi.nlm.nih.gov/compound/126941#section=2D-Structure (accessed on 17 March 2022)); Irinotecan: PubChem Identifier CID 60,838 (https://pubchem.ncbi.nlm.nih.gov/compound/60838#section=2D-Structure (accessed on 17 March 2022)); Daunorubicin: PubChem Identifier CID 30,323 (https://pubchem.ncbi.nlm.nih.gov/compound/30323#section=2D-Structure (accessed on 17 March 2022)); Doxorubicin: PubChem Identifier CID 31,703 (https://pubchem.ncbi.nlm.nih.gov/compound/31703#section=2D-Structure (accessed on 17 March 2022)); Epirubicin: PubChem Identifier CID 41,867 (https://pubchem.ncbi.nlm.nih.gov/compound/41867#section=2D-Structure (accessed on 17 March 2022)).

The following paragraphs attempt to summarize currently available data concerning occupational exposure to specific categories of ADs (Figure 1) obtained through monitoring internal doses in different biological matrices (Table 1).

### 3.1. Alkylating Drugs

#### 3.1.1. Cyclophosphamide, Ifosfamide, Bendamustine

Cyclophosphamide (CPA) is one of the most dangerous ADs, widely used for the treatment of leukemias and lymphomas, and many types of bladder, ovarian, breast, lung, endometrium, neuroblastoma, and retinoblastoma cancers [50]. Ifosfamide (IP) is an alkylating agent similar to CPA, used in the treatment of several forms of lymphomas, sarcomas, and advanced forms of solid organ tumors [51]. Different studies have found traces of these ADs in biological matrices, primarily in urine of healthcare workers, and some of these reported proportions of workers or samples with biological contamination [2,36,37,47,48].

An investigation carried out by Canal Raffin et al. [2] on French hospital professionals employed in chemotherapy reconstitution units and care services demonstrated that 23 out of 635 (3.6%) and 2 out of 357 urine samples (0.6%) were positive for CPA and IP contamination, respectively. Nurses were the most frequently contaminated category (18.2%), two pharmacy technicians were found positive to CPA, in one case following an accidental exposure, while none of the other enrolled workers showed any contamination. In oncology healthcare Iranian workers, among 60 total urine samples, 46.7% and 16.7% were positive for CPA and IP, respectively [37]. The samples of the unexposed controls had no detectable concentrations of such drugs. Interestingly, concerning the results of pre-shift and post-shift urinary monitoring, the presence of CPA and IP in 33.3% and 6.7% of pre-shift samples, respectively, suggested the possibility for an exposure occurring during the previous working day, with a possible release through the renal system in the subsequent days. Additionally, the large amount of CPA and IP in post-shift samples, 0.57 and 0.26 ng/mL, respectively, revealed unexpected exposure to the drugs during the work shift.

Compared to these investigations, a greater percentage of CPA-positive 24-h urine samples was reported in a previous study performed on healthcare workers from six British Columbia hospitals [47]. In fact, 55% of the 201 samples had detectable CPA concentrations, with a mean level of 0.156 ng/mL. No correlation between the urinary levels and known contact with CPA during the workshift could be demonstrated. In fact, unit clerks had the highest average level, and also workers engaged in the drug administration unit, but who were not responsible for administering the drugs to patients, such as volunteers, oncologists, ward aides, and dieticians, had the largest portion of samples with detectable CPA. This finding may be related to the fact that workers who did not receive training had higher levels of urinary CP concentration, thus supporting the idea that training is an important administrative control to reduce the level of occupational exposure to ADs [47].

Ramphal et al. [48] also noted a high rate of urinary CPA contamination among pharmacy staff (100% of the 7 exposed workers). However, the same percentage of positivity was detected in unexposed controls, who did not handle chemotherapy drugs (5/5), maybe due to a possible contact in the oncology pharmacy while training to use the urine-collection kit on the day of sampling. In line with this possible source of exposure, the environmental monitoring results demonstrated a widespread CPA contamination in the oncology pharmacy where chemotherapy doses were stored and prepared. Furthermore, in the second repeated series of samples, only the control subject that briefly visited such workplace area during the 24-h study period tested positive for urinary CPA. Rezazadeh Azari et al. [36], measuring the urinary CPA concentrations in the oncology personnel in two hospitals in Tehran, found that 10 out of 32 urine samples (31%) had detectable concentrations and nurses were the category with the most positive samples. Santos et al. [34] found higher levels of CPA in pharmacists and nurses with respect to unexposed controls from the hospital staff. Among the 74 French nurses investigated by Villa et al. [31], 45 reported an internal AD contamination. Among those, 37.8% and 33.3% presented internal contamination with CPA and IP, respectively, with the highest median concentration close to the LOQ of the analytical methods. Conversely to the above-mentioned results, other studies failed to detect any concentration of CPA and IP in exposed healthcare workers [10,13,32,38,40,41,44,45]. When biological monitoring was performed on two Belgian pharmacy technicians engaged in CPA preparation with the aid of a robotic system and without appreciable occupational exposure, undetectable levels of CPA could be retrieved in the 24-h urine samples [13]. In Koller et al. [41], environmental results from the oncology department of a Swedish hospital showed 50% of sampling sites with positive results for CPA, while no trace could be determined in 98 urine samples collected from 15 workers before and after their shift. Dugheri et al. [40] made the same observation. In fact, while 3.9% of samples collected from the work surfaces of the cytostatic preparation and administration units of an Italian hospital had positive results, among 398 healthcare workers with 24-h urine collection, no traces of ADs, including CPA and IP, could be detected. Comparably, Palamini et al. [32] showed no traces of CPA and IP in the 24 h-urine of 18 nurses and pharmacy technicians assigned to the hematology-oncology departments of three Canadian healthcare centers. These results corroborate those previously obtained from 101 workers at the same departments, where no detectable urinary CPA levels could be determined [44]. Concerning PPE, nurses reported wearing all the recommended protection for technical activities (86.2%), but rarely for non-technical ones (14.9%), while pharmacists and pharmacy technicians used protection for all their job tasks (100.0%). Comparably, a group of 15 Italian healthcare workers involved in the preparation, manipulation, distribution, and transport of chemotherapeutic drugs, but also cleaning of the antiblastic preparation lab, blood, and urine samples were negative in the biological monitoring for CPA, IP and Bendamustine [38], confirming the findings previously retrieved by Fabrizi et al. [45]. Furthermore, in the case of a possible acute exposure due to anticancer drug spills, no detectable levels of CPA and Bendamustine, as well as those of other ADs, could be determined in plasma samples of US healthcare workers, both after 2 and 24 h from contact [10].

#### 3.1.2. Platinum Compounds

Platinum (Pt) complexes are used to treat approximately half of all patients receiving cancer chemotherapy [52]. Cisplatin was the first Pt compound discovered to have anticancer activity. Following its introduction into the clinical management of cancer, two other Pt drugs received widespread regulatory approval, Carboplatin and Oxaliplatin. These compounds exert their antitumor effects by forming DNA adducts and subsequent inhibition of DNA replication and transcription.

A French investigation assessed the internal contamination of healthcare workers, surgeons and nurses during open abdomen heated intraperitoneal perioperative chemotherapy (HIPEC) procedures using Oxaliplatinum to treat peritoneal carcinomatosis [49]. The main drawback of this technique is the risk for leakage and contamination of healthcare workers through inhalation, as well as direct or indirect skin and eye contact. In all workers involved in the procedure, urinary Pt was undetectable. Interestingly, also no significant atmospheric contamination could be determined in this study, maybe due to the poor volatility of the Pt-containing cytostatic drugs. Conversely, a heavy contamination of the operating table, the floor at the surgeon’s feet and his overshoes could be determined during the operation, probably as the result of the drug spillage during manual supervisions of the intra-abdominal Oxaliplatinum perfusion. Similarly, when blood analysis was performed in two Danish surgeons engaged in pressurized intraperitoneal aerosol chemotherapy (PIPAC) procedures, Graversen et al. [46] could not find any contamination. The Pt analysis performed by Ndaw et al. [42] on urine samples collected from 10 volunteers of the medical staff and 5 from a control group engaged in HIPEC and PIPAC activities demonstrated levels under the LOQ for more than 50% workers’ samples, with no significant differences compared to controls. Nevertheless, environmental contamination could be detected in various locations in the operating rooms, including gloves, hands, devices, and floor. In a more recent study, Saint-Lorant et al. [28] demonstrated a Pt contamination in 36% (7 out of 19) of plasma samples collected from a French surgeon engaged in 17 HIPEC procedures for a period of 3 years.

No traces of Pt-based drugs were detected in the 24-h urine samples collected from 398 Italian healthcare workers by Dugheri et al. [40]. In Koller et al. [41], despite the considerable contamination levels determined on various surfaces on the oncology ward, the biomonitoring results of the screened staff (14 nurses, 1 doctor) were for 98% of cases in the range of the non-exposed population. In fact, most urinary Pt concentrations (96/98) were below the German reference value (10 ng/L), ranging from 0.2 to 7.3 ng/L. Interestingly, the only two nurses who had pre-shift urinary Pt concentrations of 10.3 and 16.2 ng/L reported not wearing gloves during all patient care activities, including washing patients and changing bed linen, or during unpacking and preparing the AD infusions. Overall, these results can exclude heavy or moderate internal Pt contamination in exposed healthcare workers, but cannot preclude very slight contamination.

Apart from blood and urine, hair samples have also been explored as a suitable matrix to investigate the Pt internal contamination in exposed healthcare workers [35]. In fact, Pt concentrations in hair from personnel handling Pt compounds were significantly higher than those found in workers non handling such compounds. However, caution should be paid in interpreting Pt biomonitoring data, as other sources of exposure, such as dental appliances, metallic Pt dust, and catalysts in car exhaust systems may be responsible for the detected levels, particularly for the comparable concentrations found in office workers and non-Pt users in the hospital.

### 3.2. Topoisomerase Inhibitors

#### 3.2.1. Irinotecan

Irinotecan (IRT) is a potent inhibitor of topoisomerase and has been used as a first- or second-line AD in several malignancies, especially for colorectal cancer [53]. Irinotecan is a good marker for the assessment of healthcare occupational exposure, as it is absent from the environment aside from healthcare settings, and has a longer half-life, which is compatible with easy biomonitoring [54].

A French study conducted by Benoist et al. [30] performed the first evaluation of blood contamination by IRT and its metabolites, 7-ethyl-10-hydroxycamptothecin (SN-38) and 7-ethyl-[4-N-(5-aminopentanoic-acid)-1-piperidino]carbonyloxycamptothecin (APC), in a centralized AD pharmaceutical unit before and after protective equipment changes. A total of 15/36 (41.6%) assays were positive before and 16/72 (22.2%) after changing collective and PPE, with the reduction in the percentages supporting the effectiveness of such measures in controlling the exposure. In a subsequent investigation, Bechet et al. [29] demonstrated that 17 out of 78 workers (21.8%) in a pharmaceutical unit of a French cancer center had detectable levels of IRT or its metabolites in plasma and red blood cells. The number of positive assays was found to be significantly higher in the staff members working outside the compounding unit compared to those operating inside, and also with respect to caregivers directly involved in AD handling.

Izzo et al. [38] monitored a group of Italian healthcare workers engaged in the preparation of antiblastic therapies. They found traces of IRT in both plasma (68 pg/mL) and urine (35 pg/mL) of one transporter/cleaner and in the plasma of one preparator (55 pg/mL). Although a quite long plasmatic half-life has been reported for this compound [55], the detection of IRT in plasma suggested that the uptake of this AD by the two workers occurred a few hours before the sample collection. However, as traces of IRT were also detected in the urine sample of one of the analyzed subjects, chronic or prolonged exposure cannot be ruled out.

When the IRT contamination was investigated in a surgeon exposed during HIPEC procedures [28], despite collective and PPE, 79% and 63% of plasma and red blood cell samples were contaminated, both soon after HIPEC procedures, as well as following a period of inactivity.

#### 3.2.2. Anthracycline ADs

Daunorubicin (DNR), doxorubicin (DXR) and epirubicin (EPI) are anthracycline ADs which can intercalate between DNA bases or generate free radicals and can also interact with cellular membranes and inhibit the nuclear enzyme topoisomerase II [56]. Doxorubicin and EPI are prescribed in the treatment of various cancers, including breast, digestive, hematological, bronchopulmonary, and ovarian cancers. Daunorubicin is used in the treatment of hematological cancers [33]. Villa et al. [33] monitored more than 77 workers for anthracycline AD exposure. Two healthcare professionals were found to be contaminated by anthracycline drugs which represents the 2.6% of the monitored population. Specifically focusing on the collected samples, one from a nurse and one from an assistant nurse were found positive to DXR or EPI with a urinary concentration level of 218 ng/L and 17.7 ng/L, respectively. Both workers reported to irregularly wear gloves. Two other studies failed to demonstrate any case of contamination by EPI in urine [45] and by DXR and DNR in blood of healthcare workers [38].

### 3.3. Folic Acid Antagonists

Methotrexate (MTX), a structural analogue of folic acid, is one of the most effective and extensively used drugs for treating many kinds of cancer or severe and resistant forms of autoimmune diseases [57]. Among the 116 subjects engaged in handling ADs investigated by Canal Raffin et al. [2], 1 caregiver was found contaminated with MTX. Among the 357 urine samples analyzed, 3 were positive with a median concentration of 36.3 pg/mL. In Villa et al. [31], 42.2% of 45 nurses with AD internal contamination were positive for MTX, with the highest median level close to the LOQ of 2.5 ng/L.

Palamini et al. [32] demonstrated the absence of MTX in the 24-h urine samples of 18 healthcare workers exposed to four ADs (CPA, IP, MTX and 5-Fluorouracil (5-FU). Similarly, in 101 workers exposed to these four drugs, no urine sample had detectable concentrations of any of the compounds evaluated [44]. Negative biological monitoring results were determined for MTX in the group of 15 healthcare workers investigated by Izzo et al. [38]. Fabrizi et al. [45] monitored 9 workers from an Italian hospital exposed to different chemotherapies including CPA, EPI, etoposide, 5-FU, Gemcitabine and Taxol in variable amounts and not at the same time. Unexpectedly, among the urine samples collected at the end of their shift, one urine sample demonstrated a high concentration of MTX (0.22 ng/mL, about 5 LOQ). The outcome seemed surprising, but was possibly due to an omission in the declaration of substances to which workers were exposed.

### 3.4. Pyrimidine Antimetabolites

The antimetabolite, 5-FU, continues to be used in the treatment of breast, gastrointestinal, head and neck, and ovarian cancers two decades after its synthesis [58]. Following a catabolic pathway, 5-FU generates alfa-fluoro-beta-alanine (FBAL) that represents its major metabolite in urine [59]. Sottani et al. [39] investigated healthcare occupational exposure to 5-FU, analyzing the concentration of its FBAL metabolite in the urine. They showed that 2 out of 77 samples, collected from pharmacists involved in the compounding of ADs and workers engaged in their administration, were found positive for FBAL, with the highest concentration of 1.8 ng/mL. The authors suggested a possible environmental contamination, as 5-FU was detected in many workplace surfaces and on the gloves of a technician involved in the compounding of this drug. Among the 45 nurses with an internal AD contamination investigated by Villa et al. [31], 17.8% presented positive results for FBAL, with the highest median concentration of 41.5 ng/L. When 73 French and African healthcare professionals were examined for 5-FU contamination through the analysis of the FBAL urinary content, 9.6% presented results above the LOQ [43]. Regarding the job categories, 5 nurses, 1 assistant nurse and 1 cleaning person were positive from the biological monitoring investigation. The highest measured FBAL concentration (301 pg/mL) was retrieved from a nurse working in a developing country. The 3 French contaminated healthcare professionals had urinary levels ranging from 25 to 35 pg/mL, values close to the analytical LOQ employed in this study.

Koller et al. [41] observed the absence of any trace of 5-FU in workers’ urine samples collected before and after their shift, although 100% of the examined surfaces of the oncology ward, including areas dedicated to the pre-administration, administration/patients’ care and handling of patients’ excreta, were contaminated by 5-FU. Poupeau et al. [44] and Palamini et al. [32], in two different studies conducted at the same hospital, showed the absence of traces of hazardous drugs in the urine of 101 and 18 healthcare workers exposed to 4 ADs including 5-FU, respectively. Dugheri et al. [40] made the same observation: among 398 healthcare workers with 24-h urine collection, no traces of FBAL were detected in the urine samples, even though 3.9% of work surfaces examined during the same period had positive results. Similarly, Fabrizi et al. [45] failed to demonstrate 5-FU contamination in a single urine sample collected at the end of the shift of 9 workers engaged in the manipulation of the drug in an Italian hospital.

### 3.5. Other ADs

In order to understand the role of AD spills in affecting biological monitoring results, Friese et al. [4] have demonstrated low, but quantifiable, levels of etoposide, docetaxel and pemetrexed in healthcare workers soon after a spill of such ADs. However, the exposure was not limited exclusively to drug spills, as also urine samples from cancer center employees who did not report a drug spill had detectable, but not quantifiable, levels of docetaxel. The contamination of surfaces in the infusion area may be the primary source of exposure. The findings from those who did and did not report a drug spill suggested that this latter occurrence could pose a greater exposure risk to healthcare workers than routine environmental exposure. Fabrizi et al. [45], in 9 workers from an Italian hospital who had manipulated or were exposed to etoposide, gemcitabine and paclitaxel in variable amounts found two cases in which urinary concentrations of taxol and etoposide were between LOD and LOQ, while no traces of paclitaxel were detected in both plasma and urine of the 15 healthcare workers studied by Izzo et al. [38].

## 4. Discussion

This review attempts to provide an overview of the most recent data on AD exposure in occupational settings assessed through biological monitoring, in order to identify also specific tasks and job procedures at increased risk of exposure. This may reveal helpful information to evaluate the effectiveness of the currently adopted measures to control the exposure and define suitable updated strategies for risk assessment and management in different occupational settings, including suitable biological monitoring programs, and risk communication strategies, to ensure the safety and health of involved workers.

Several studies have demonstrated that workers exposed to ADs may have traces in their biological fluids. Indeed, despite the existence of suitable work practices and control measures, some portions of workers are at risk of AD exposure. However, a great deal of variability with respect both to AD concentrations (when available), and percentages of positive samples emerged. Some studies reported no drugs determined [10,13,32,38,39,40,41,44,45], while others demonstrated that up to 55% of the analyzed urine samples exceeded the LOD [2,30,35,36,37,47,48]. These results are in line with those retrieved in previous reviews reporting that, even when technical and PPE was used, a remarkable uptake of ADs could be observed, as percentages of positive urinary samples ranged from 0% to 88.9% in the hospital personnel [21]. A more recent analysis found a percentage of positive urine samples around 30% in the 1990s and 2% in the 2000s, with no positive samples detected in 2006 or 2007 [60]. Suspiro and Prista [61], on the other side, reported that, in the majority of the reviewed studies, measurable levels of the cytostatic drugs or their metabolites were detected in urine samples from exposed workers, indicating that significant absorption occurred in most work situations.

The differences within and between studies may be likely due to variations in the extent of AD use (as the risk of contamination is greater according to a longer use of a particular drug or to a greater number of operations performed), variable handling and cleaning practices, the occurrence of spills, collective and PPE adopted, as well as to the variability in metabolic rates among individuals [47,62]. Concerning a time-related trend, in line with the above-mentioned reviews [60,61], decreasing percentages of CPA- and IP-positive urine samples were detected in the 2015–2021 examined period, with more recent studies failing to determine AD concentrations in biological samples, maybe in relation to improved work practices [32,38,40,41,44]. Negative biological samples, however, should not be assumed to represent an absence of risk, since even a very low exposure level can theoretically be associated with genotoxic effects, and also the sensitivity of the analytical methods used from measurement is a critical issue to be considered [61]. A greater number of investigations assessing various substances on larger sample sizes and enrolling unexposed subjects as controls seem necessary to extrapolate definite conclusions in this regard, in order also to assess the effectiveness of the measures adopted in these latter years to control workplace exposures.

In addition to these already mentioned factors, which may explain differences in biological contamination, analytical variabilities cannot be ruled out. Most of the retrieved studies, in fact, primarily attempted to develop analytical methods to detect several drugs in the biological matrices, more than to assess healthcare professional exposure. In this view, sensitive, specific, and standardized analytical methods are still needed to define the effective biological exposure to combinations of several antiblastic drugs [38]. The development of selective, efficient, and sensitive techniques should be strongly encouraged in order to drastically reduce pre-analytical procedures, times and costs of the whole process, and to minimize the risk of artefacts, while assuring a routine application to guarantee workers’ safety and health. In this perspective, given that the investigated substances represent only a limited portion of the dozen of chemotherapies that healthcare workers routinely prepare and administer in their hospitals, and considering their dangerous toxicological profile, to define analytical strategies to assess occupational exposure through suitable biomarkers should become a priority of healthcare institutions. This is also confirmed by the meta-analysis of Roussel et al. [63] who found a significant association between occupational exposure to ADs during the course of a normal work day and increases in chromosomal aberrations in healthcare workers. Thus, an appropriate methodology for biological monitoring should be focused neither on a single molecule as a biomarker of internal dose, nor on a single biological matrix.

Human exposure to chemicals was mainly assessed using either urine or blood, whose analysis showed both advantages and drawbacks [38]. Nonpersistent chemicals, such as ADs, usually have short half-lives in blood, where their concentration after each exposure rapidly declines. Measurements of drug metabolites in urine allowed a much wider window of opportunity to analyze the sample, although the exposure to multiple ADs in occupational settings, urine dilution, and specificity issues often complicate the analysis [64]. Some studies attempted to overcome such difficulties through the collection of urine over a 24-h period [65,66]. Multiple matrix types should be considered for drug biomonitoring in order also to find more specific and sensitive biomarkers and define possible associations between indicators according to a deeper understanding of the kinetics of the parent drugs and their metabolites [29]. In this perspective, hair samples may offer advantages over blood and urine testing including a less invasive nature, the easy storage and long survival time of the samples, and the ability to determine exposure history [35]. However, the shortage of biomonitoring data on this matrix prevents suitable conclusions on its applicability in occupational contexts. Multiple sampling time, including pre- and post-shift analyses, may be also considered in order to extrapolate data useful to inform suitable biological monitoring plans. Additionally, in order to overcome discrepancies between populations and obtain more homogenous results to be compared, it may be desirable to have standardized operative procedures to assure sample traceability, proper collection, transfer and storage of samples, as well as homogeneous analyses.

In regard to the investigated populations, in the literature, these were mainly selected among staff members considered at risk of exposure, particularly staff whose work consisted of handling ADs, in compounding or administration phases, i.e., nurses, pharmacy technicians, oncology healthcare workers, that resulted also in the most frequently contaminated categories [2,36,37]. However, in some studies, participants from departments/areas where drug preparation and administration did not take place (shipping/receiving, transport, nutrition, and materials management) had also detectable [29,48] and, in some cases, higher average drug concentration levels [47] than those workers directly involved in preparing/handling/administering chemotherapy. Overall, these results support the idea that other workers within the hospital medication system, besides nurses and pharmacy personnel, may be at risk of exposure to ADs [67]. Despite this evidence, very few studies have aimed to assess environmental and biological contamination “outside” directly involved antineoplastic areas. This prevents a great number of workers, such as pharmaceutical staff working outside the compounding unit, but also members of the oncology care units who are poorly exposed to ADs, administrative and transport staff, cleaning employees, and unit clerks, from developing awareness of the AD issue and establishing suitable protective measures [47,67].

Additionally, the variability in reporting results and the quite fragmented data extrapolated limit the interpretation of the results. The revised studies, in fact, in some cases included percentages of positive workers or positive samples, mean and/or median levels of ADs detected in exposed and control workers, the indication for positive results to one or more ADs simultaneously, without details specifically focused on single compounds, as well as the not always clear levels of exposure of specific job categories, in relation to acute (accidental) or chronic conditions of exposure. Overall, this prevents extrapolation into definite conclusions on qualitative and quantitative types of exposure per job category or suitable comparisons and needs to be overcome in future investigations. Moreover, the increasing application of ADs in innovative surgical procedures, such as the HIPEC and PIPAC, as well as the employment of such drugs also for non-malignant diseases, may increase the likelihood for healthcare occupational exposure [28,36,42]. Particularly, the exposure for healthcare workers to ADs during open abdomen HIPEC is a subject of concern since healthcare workers in the operating room generally have no experience in handling these drugs. Cytostatic drugs are heated before administration, which facilitates their vaporization, and the open technique implies manual control of the distribution of the chemotherapy solution in the abdomen, with the associated risks of splashes and direct contamination of the surgeon [42,49].

All these elements raise questions regarding which workers need to be monitored for AD exposure and point out the relevance to evaluate the association between environmental and biological monitoring data. In this view, few studies of those included in our review performed environmental and biological monitoring analyses. However, it may be useful to collect environmental samples concurrently with biological ones to achieve complementary “information on contamination”. The former, in fact, can demonstrate how, where, and possibly when contamination occurred, while the latter could indicate if exposure occurred in workers [21]. The relevance of a deep environmental analysis relies also on the evaluation of the efficacy and safety of collective equipment, including engineered enclosed systems, in reducing environmental exposure and consequently internal doses of exposed workers [13,17,30]. This issue needs a careful assessment as recent data reported no evidence of differences in the proportion of people with positive urine tests for exposure between those employing closed-system drug-transfer devices and safe handling of ADs and the control groups adopting only safe handling procedures for CPA alone; CPA and IP; or CPA, IP and gemcitabine [68]. Moreover, information on the possible co-exposure to other chemical or physical carcinogenic factors, including ionizing radiations, may also be derived from risk assessment analyses and may be helpful to understand possible early biological alterations in exposed workers, and to adopt suitable health surveillance and health promotion plans [69].

However, a workplace study is also helpful in identifying the most relevant urinary biomarkers of ADs to monitor as well as the most suitable timing for sampling. This choice, in fact, should be the result of a complex analysis addressing the nature, danger, frequency, amount of handled drug in healthcare departments, and the most sensitive analytical methods available [2,70]. Additionally, exposure by inhalation, in fact, may result in a very quick uptake and excretion in the urine, while exposure via the skin can cause a postponed excretion over a longer period, maybe in relation to the barrier function of the skin. Currently, a suitable risk assessment for healthcare professionals occupationally exposed to ADs is prevented by the lack of biological limit values (BLVs) or biological guidance values (BGVs) [33]. Moreover, the health impact of low-dose internal contamination level of ADs is not currently known and the concentrations retrieved in biological matrices cannot be easily interpreted. Bearing in mind the carcinogenic and reprotoxic effect of these drugs, the ALARA principle “As Low-level as Reasonably Achievable” should be adopted [71]. Additionally, for a correct interpretation of biological monitoring results, information on drug handling (nature, frequency and quantity), performed tasks, collective control systems used, wearing of PPE, and industrial hygiene practices, should be carefully collected. In general, the presence of ADs in biological matrices makes it possible to establish the occurrence of an internal contamination of the worker and should be considered as a failure of preventive measures, therefore requiring corrective interventions from all the preventive figures engaged in occupational health and safety.

Apart from duties involving handling ADs, whether the workers had ever received safe drug handling training as well as the adoption of collective and PPE can affect biomonitoring results. The relevance of training as an administrative control to reduce the level of occupational exposure to ADs is also supported by the fact that controls, while having a much shorter duration of exposure to the contaminated environment than the oncology personnel, showed greater biological exposure to CPA, maybe due to the fact that the latter may have been more vigilant about hygiene practices and using protective measures [47]. However, further evaluation of the role of training and the one’s level of knowledge related to the risks of ADs with respect to the effectiveness in reducing the risk of exposure is necessary. In this perspective, possible barriers to the compliance and safe handling of ADs, including poor training, poor safety culture, and inconsistent policies, and common facilitators, such as adequate safety training, leadership support, and consistent policies, should be deeply assessed as administrative aspects of preventive intervention [72]. Although interesting, the present review has some limitations that should be noted. First, the number of healthcare professionals enrolled in the sampling investigations was rather small, which limits the generalizability of the findings. Indeed, future investigations should confirm the retrieved findings on larger groups of “directly and indirectly” exposed workers. As the biological monitoring findings are only representative of the point in time when samples were collected, multicenter studies and follow-up, longitudinal investigations would also be necessary to establish the exposure levels of workers, also in different conditions and pressure of work and changing work and protective equipment. It should also consider that staff engaged in antineoplastic handling in some small facilities often have multitasking activities both in compounding/administration areas as well as in other departments of the workplace. This is yet another confirmation of the need to provide a specific safety program to the “whole” antineoplastic involved staff, including a regular environmental and biological monitoring. Moreover, studies should always audit for the collective and PPE employed during the study period. Research efforts should focus on intervention development and evaluation, multisite studies to compare exposures, also considering organizational factors, and studies that correlate exposure to health outcomes.

## 5. Conclusions

This review provides un updated overview on the biological monitoring of occupational AD exposure in order to extrapolate information useful to improve risk assessment and management strategies in workplaces. In fact, despite the adoption of preventive and protective measures, variable percentages of healthcare workers may have positive results at the biological monitoring analyses, with nurses as the job category at increased risk of exposure. However, further investigations seem necessary to deeply understand those factors that may affect the internal doses, such as the extent of ADs use, work procedures, acute or chronic contacts, training of the workforce, as well as the adoption of collective and PPE. In this still formative phase of knowledge, these approaches will generate the necessary evidence to address a 30-year-old problem and collaborative efforts will provide the basis to improve optimal strategies to protect workers while maintaining the clinical efficiency of antineoplastic therapies.

## Figures and Tables

**Table 1:** Studies assessing occupational exposure to ADs through biological monitoring.

References	Occupational Setting/Number of Workers/Time Period of the Study	Analytical Method/Biomarkers			Outcome	Results	Quality Rating (Numerical Score)
		Investigated Antineoplastic Drug	Biomarker of Exposure/Matrix/Sampling Time	Method LOD and LOQ			
Canal Raffin et al. [2]	Ten centralized chemotherapy reconstitution units and eight care services from 11 French hospitals/116 healthcare workers: 48 PTs, 44 nurses, and 24 other employees (i.e., stretcher bearers, patient area cleaners, caregivers, and healthcare assistants). Time period: NA	✓ CPA ✓ IP ✓ MTX	CPA, IP and MTX/urine (635 samples for CPA; 357 for IP and MTX)/samples were collected one before the shift and one after a working day	ESI-LC-MS/MS with liquid/liquid for CPA, IP and solid phase extraction for MTX LOD (pg/mL): 10 for CPA, IP, MTX LOQ (pg/mL): 20, for CPA, IP, MTX	To develop and validate highly sensitive, specific and reliable analytical tools for CPA, IP, and MTX detection in urine	A total of 28 urine samples were positive to at least one of the 3 investigated drugs (11 workers, 9.5% of the population). Among the 23 CPA positive urine samples, 6 showed concentrations at a trace non-detectable level (above the LOD, but lower than LOQ). Median concentration for CPA was 40.7 pg/mL with values ranging from 20.1 to 1850 pg/mL. The concentrations determined for IP were 25 and 37 pg/mL.	Good (7)
Saint-Lorant et al. [28]	A comprehensive cancer centre in France/A surgeon engaged in 17 HIPEC procedures in the investigated period. Time period: September 2015–April 2018	IRT and its metabolites (SN-38, APC)	IRT, SN-38, APC, Pt/19 blood samples collected from the surgeon	UHPLC for IRT and its metabolites; ICP-MS for Pt compounds LOQ (pg/mL): IRT 50; Pt 16	To assess levels of IRT and Pt in an exposed surgeon	IRT contamination in plasma: 15 out of 19 samples (79%). Minimum (92 pg/mL) and maximum (266 pg/mL) quantified concentration (13/19 samples). IRT contamination in RBCs: 12 out 19 samples (63%). It was quantified in 4 (21%) out of 19 RBC samples with a minimum and a maximum of 114 and 257 pg/mL. SN-38 contamination: 4 and 9 out of 19 plasma and RBC samples, respectively. No APC detected in plasma. Pt compound contamination: 7 out 19 samples.	Unsatisfactory (4)
Béchet et al. [29]	Pharmaceutical unit of a French comprehensive cancer centre/7 PTs, 4 pharmacists and 2 pharmacy students (sex ratio M/F: 0.6; median age: 38 years). Time period: NA	✓ IRT	IRT and its metabolites (SN-38; APC)/plasma and red blood/sampling was performed within a time frame of 27 to 31 h after a possible IRT manipulation	UHPLC-MS/MS. LOD pg/mL:(2.5) LOQ (pg/mL): (50)	To assess blood contamination by IRT and its metabolites in the pharmaceutical staff working inside and outside a compounding unit.	A total of 17/78 (21.8%) plasma and RBC-based assays were found to be contaminated among the investigated staff. Positive assays were higher in the staff members working outside the compounding unit (5/42; 11.9%) than for workers working inside (12/36; 33.3%) (*p* = 0.022).	Unsatisfactory (5)
Benoist et al. [30]	French university hospital/8 PTs, 2 pharmacists and 2 cleaning agents (sex ratio M/F: 0.2; median age: 38 years); the average duration of worker exposure was 7 h per day. Time period: NA	✓ IRT	IRT and its metabolites (SN38 and APC)/plasma and red blood/sampling was performed within a time frame of 27 to 31 h after a possible IRT manipulation	UHPLC-MS/MS LOD pg/mL:(2.5) LOQ (pg/mL):(50)	To assess blood contamination with IRT and its metabolites for cytotoxic drug preparations personnel before and after equipment changes	A total of 15/36 (41.6%) assays were positive (>LOD) before equipment changes; 16/72 (22.2%) after equipment changes, with a significant decrease between periods (*p* = 0.035).	Satisfactory (6)
Villa et al. [31]	Two French hospitals/nurses (74) who worked on average 3.9 ± 1.4 days prior to the day of the study and 79.7% declared to be exposed at least once to at least one of the 5 ADs investigated.	✓ CPA ✓ FBAL ✓ IP ✓ MTX ✓ 5-FU ✓ DXR	CPA, FBAL, IP, MTX, 5-FU, DXR/urine/ samples were collected within the 3 h before the start of the work, within 2 h from the end of the work shift, between 7 and 10 h after the end of the work shift	UHPLC-MS/MS LOD ng/L: 1, for CPA, IP and MTX; 5 for DXR and 14 for FBAL. Lower LOQ (ng/L): 2.5–20 for CPA, IP and MTX; 10 for DXR and 20 for FBAL	To determine the concentration of the 5 ADs in exposed workers at different timings	Internal contamination by at least one of the 5 ADs was found in 60.8% of nurses (45/74). Regarding nurses with internal contamination, 42.2% presented internal contamination by MTX,37.8% by CPA, 33.3% by IP, 17.8% by 5-FU metabolite and 6.7% by DXR. The highest median concentrations were obtained for DXR (232.0 ng/L) and FBAL (41.5 ng/L). For IF, CP and MTX, the median concentrations were close to the LOQ (2.5 ng/L) of the corresponding methods.	Satisfactory [6]
Palamini et al. [32]	Hematology-oncology departments of 3 healthcare centers in the region of Montreal, Quebec, Canada/18 healthcare workers (10 nurses and 8 technicians, age range 20–50 years; mean work history: 7.7 ± 9.6 and 7.8 ± 5.0 years for nurses and PTs, respectively) who worked at least the two days immediately before the 24-h sampling period. Time period: 1–30 September 2019	✓ CPA ✓ IP ✓ MTX ✓ 5-FU	CPA, IP, MTX, FBAL/urine/24 h urine samples	UHPLC-MS/MS LOD (pg/mL): CPA (9.0) IP (9.7) MTX (75) FBAL (120)	To determine the concentration of the 4 hazardous drugs in workers’ 24-h urine samples	No traces of CPA, IP, MTX or FBAL were found in the 24-h urine samples (128) collected from the 18 healthcare workers	Good (7)
Villa et al. [33]	Nine hospitals including 8 French hospitals and 1 non-French from an African country/77 healthcare workers occupationally exposed to anthracyclines (29 nurses, 10 cleaning persons, 18 assistant nurses, 13 PTs, 2 pharmacists) Time period: NA	✓ DXR ✓ EPI ✓ DNR	DXR, EPI, DNR/urine/spot samples collected 7–10 h after shift of one or several working days of exposure	UPLC/MS-MS LOD (ng/mL): DNR 0.001; EPI 0.0025; DXR 0.005 LOQ (ng/mL): DNR 0.010; DXR 0.010; EPI 0.1	To develop a suitable method to determine anthracycline concentrations in the urine samples of healthcare workers	Two healthcare professionals (2.6%) from the non-French hospital were found to be contaminated to DXR and/or EPI. Urinary concentration levels for DXR and EPI was, respectively, 218 ng/L and 17.7 ng/L.	Satisfactory (6)
Santos et al. [34]	One Brazilian hospital/pharmacists (25), nurses (24), unexposed controls (10) with a minimum weekly workload of 20 h with >4 months of exposure. Time period: December 2017–February 2017.	✓ CPA	CPA, IP/urine/samples were collected on Friday afternoon at the end of the week work shift.	GC/MS LOD (ng/mL): 0.03 and 0.11 for CPA and N-trifluoroacetylated CP.	To determine the CPA concentrations in urine of exposed workers compared to controls	The presence of CPA and/or its metabolites was 6 and 6.5-fold increased in pharmacists and nurses, respectively	Unsatisfactory (5)
Hori et al. [35]	Five departments of the Center Hospital of the National Center for Global Health and Medicine, Tokyo/doctors, nurses and pharmacists from the hematology, respiratory and gastroenterology departments, a diabetes ward and pharmacy (13 M and 46 F in 2010, age 22–49 years; 24 M and 52 F, age 23–60 years in 2015). Non medical office workers (15) enrolled in 2015 as controls. Time period: July 2010 and April 2015	✓ Pt	Pt/hair samples	LA-ICP-MS LOQ (ng/mL): 0.001411 in 2010; 0.001272 in 2015	To determine the Pt concentration in hair samples of healthcare exposed workers	Median Pt levels (×10^−3^ng) in hospital workers (2010–2015): Pt users (37), 3.14 (interquartile range 2.35–4.42); non users (48), 2.51 (interquartile range 1.61–4.74). Median Pt levels (×10^−3^ng) in office workers: 2.17 (interquartile range 1.62–2.85) Median Pt levels (×10^−3^ng) in treated patients (15): 213.16 (interquartile range 31.90–627.25).	Good (8)
Shu et al. [10]	Twelve cancer centers in the USA/participants from the centers (378; 64 experienced drug spills)	✓ PTX ✓ DXR ✓ Etoposide ✓ Gemcitabine ✓ Bendamustine ✓ Docetaxel ✓ Irinotecan ✓ CPA ✓ Other drugs	Anticancer drugs (18)/plasma samples (743)/samples were collected at baseline, after the educational assessment and whenever they experienced a drug spill (at 2 and 24 h from the spill)	MRM-IDA-EPI LLOD (ng/mL): 0.10–1.0 LLOQ (ng/mL): 0.10–1.0	To develop a method to assess the plasma concentration of 18 ADs in acute exposures	All plasma sample measurements were below the lower LOD at baseline, post-intervention, and in cases of documented acute spills	Good (7)
Rezazadeh Azari et al. [36]	Two hospitals in Tehran (Iran)/Oncology personnel (45) as PTs, nurses, and auxiliary workers (Mean age: 29.75 years; Mean work history: 3.12 years) Time period: September 2015–January 2016	✓ CPA	CPA/urine/samples collected at the end of the work shift	GC-ECD and GC-MS (as confirm) Lower LOD (ng/mL): (0.2) Lower LOQ (ng/mL): (0.5)	To validate a method for analysing CPA in urine samples	Urinary CPA concentrations were between 0.52 and 21.4 g/L in the urine of 31% of two hospital staff. Mean CPA concentration in the two hospitals: 9.53 ± 7.33 and 11.98 ± 9.75 ng/mL	Unsatisfactory (5)
Baniasadi et al. [37]	An oncology teaching hospital in Iran/healthcare workers (15): 9 nurses, 3 nurse assistants, 2 cleaners, 1 secretor (mean age: 31.13 ± 6.45 years; mean work history: 1 year; male/female 6/9); non exposed personnel as a controls (15) (mean age: 37 ± 6.16 years; mean work history: 0 year; male/female 5/10). Time period: NA	✓ CPA ✓ IP	CPA and IP/urine/samples were collected in pre and post shift	GC/MS LOQ (ng/mL): CPA (0.04) IP (0.05)	Determine CPA and IP concentrations in urine samples of exposed workers	CPA was detected in 5 pre-shift and 9 post-shift urine samples. One pre-shift and 4 post-shift urine samples were positive for IP Mean CPA concentration in post-shift samples: 0.57 ng/mL (range 0.22–1.04) Mean IP concentration: 0.26 ng/mL (range: 0.12–0.35)	Satisfactory (6)
Izzo et al. [38]	University Hospital in Salerno (Italy)/15 healthcare workers involved in the preparation, manipulation, distribution, transport of chemotherapeutics and in the AD lab cleaning Time period: NA	✓ MTX ✓ CPA ✓ IP ✓ IRT ✓ DXR ✓ DNR ✓ Bendamustine ✓ PTX	MTX, CPA, IP, IRT, DXR, DNR, BMA, PTX/plasma and 24-h urine/ samples were collected at the end of the working day, during the last day of working week.	UHPLC-MS/MS Lower LOD (pg/mL) range: 2.5–15 and 2.5–5 in plasma and urine, respectively Lower LOQ (pg/mL) range: 5–15 in both matrices (50 pg/mL for PTX)	To develop, optimize and validate a novel UHPLC-MS/MS method for the simultaneous quasi-quantitative analysis of a panel of antineoplastic drugs	Thirteen out of 15 workers were negative to the biological monitoring. Traces of IRT were detected in both plasma (68 pg/mL) and urine (35 pg/mL) of one transporter/cleaner and, at a lower level (55 pg/mL), in the plasma of one preparator.	Unsatisfactory (4)
Sottani et al. [39]	Eight hospitals/healthcare workers (38, urine samples: 20 from pharmacists involved in the compounding of ADs and 57 from workers who administered such drugs) Time period: NA	✓ Cape ✓ 5-FU	FBAL/urine/ sampling was performe at the pre and post shift work (7 h after the beginning of the activities)	rp-UHPLC-MS/MS LOQ (ng/mL): 0.5	To measure the urinary (pre and post-shift) excretion of FBAL in healthcare workers involved in the compounding of antineoplastic drugs and operating in administering units	Two urine samples out of 77 were found positive for FBAL (the highest concentration for FBAL was 1.8 ng/mL)	Good (7)
Dugheri et al. [40]	Careggi University Hospital, Florence/398 healthcare workers (nurses, technicians, and pharmacists) who handled ADs at the same time as when the wipe samples were collected. Time period: 2009–2017	✓ CPA ✓ IP ✓ Pt compounds (cis-, carbo-, and oxali-Pt) ✓ 5-FU	CPA, IP, Pt, and FBAL/urine/ samples collected before AD administration or preparation and until the next day	LC/MS-MS and ICP-MS (for Pt) LOD (ng/mL): CP 8.1; IP 7.7; Pt 15.4; FBAL 234. LOQ (ng/mL): CP 25.3; IP 22.9; Pt 46.2; FBAL 643.	To evaluate the contamination of work areas though environmentaland biological monitoring	No urine sample had detectable concentrations of any of the 4 drugs considered (0/398 samples).	Good (7)
Koller et al. [41]	A hospital in Southern Germany/15 health care workers from the oncology department (13 female and 1 male nurses and 1 female physician) Average age: 38 years; average time of ADs handling experience: 8.7 years. Time period: July 2017	✓ 5-FU ✓ CPA ✓ Pt compounds (cis-, carbo-, and oxali-platin)	CP, Pt, and FBAL/urine/samples collected before and after daily shift for an average of 3.5 days	GC/MSMS LOD (ng/L): CP 0.05; FBAL 0.2; Pt 0.001	To assess the occupational exposure of oncology ward employees to ADs by a combination of environmental and biological monitoring	No FBAL or CP residues were detected in any urine sample Regarding Pt analysis, most urinary Pt concentrations (96/98) were below the German reference value (10 ng/L). Two nurses had pre-shift urine Pt concentrations of 10.3 and 16.2 ng/L.	Unsatisfactory (5)
Ndaw et al. [42]	A French Hospital, department of digestive and oncologic surgery/medical staff performing HIPEC (5) and PIPAC (5) procedures, control group included unexposed medical personnel (5).	✓ Cisplatin	Pt/urine/24-h urine samples were collected from the void in the morning before the procedure (32 and 23 for HIPEC and PIPAC procedures); pre-shift and post-shift samples (18) were collected from controls during two consecutive days	ICP-MS LOQ (ng/mL): 10	To assess occupational exposure to Pt during HIPEC and PIPAC procedures	Controls: 72% samples above the LOQ (range: <LOQ-91 ng/L, median concentration: 12 ng/L) HIPEC procedures: 44% samples above the LOQ (range: <LOQ-87 ng/L; median concentration: <LOQ). No significant differences with the controls PIPAC procedures: 48% samples above the LOQ (range: <LOQ-136 ng/L; median concentration: <LOQ). No significant difference with controls and HIP	Good (7)
Dhersin et al. [43]	Eight French hospitals including one from the African country/Health care professionals (73:48 nurses, 15 cleaning staff, 7 assistant nurses, 3 PTs)	✓ FBAL	FBAL/urine/spot samples collect from 0 to 10 h after the work shift.	ESI-UHPLC/MS-MS		Seven urine samples from 73 were positive for healthcare professionals (9.6%).	Satisfactory (6)
Poupeau et al. [44]	A mother–child university health center in Quebec, Canada/92 workers from the hematology–oncology department (74 nurses, 5 pharmacists, 6 PTs, 7 doctors) and 9 participants not working in hematology–oncology as controls (6 pharmacists, 3 PTs). Mean age of experience: 6.5 ± 2.1, 8.3 ± 10.1, 13.3 ± 11.8 and 16.0 ± 13.3 years for nurses, pharmacists, PTs, and doctors, respectively. Time period: 15–29 January 2015	✓ CPA ✓ IP ✓ MTX ✓ 5-FU	CPA, IP, MTX, FBAL/urine/one spot urine sample was collected at the end of the work shift	UPLC/MS-MS LOD (pg/mL): CPA 9.0; IP 9.7;MTX 75; FBAL 120 LOQ (ng/mL): CPA 30; IP 32; MTX 250; FBAL 400	To determine the concentration of four ADs in urine samples of healthcare workers	No urine sample had detectable concentrations of any of the four drugs evaluated	Good (7)
Fabrizi et al. [45]	An Italian hospital/nine healthcare workers (nurses, a health care assistant, a pharmacist, a head nurse and a front desk officer) Time period: NA	✓ CPA ✓ EPI ✓ VP-16 ✓ 5-FU ✓ GCA ✓ PTX	CPA, EPI, VP-16, 5-FU, GCA and PTX/urine/single urine sample collected at the end of shift	UPLC/MS-MS LOD (ng/mL): CPA 0.33; EPI 0.03; VP-16 0.17; 5-FU 33.33; GCA 0.67; PTX 0.33. LOQ ng/mL: CPA 1.00; EPI 0.10; VP-16 0.50; 5-FU 100.00; GCA 2.00; TAX 1.00	To develop a fast and easy tailored dispersive solid-phase extraction procedure for determination of 13 cytostatic drugs	Two samples demonstrated a taxol and VP-16 concentration between LOD and LOQ.	Unsatisfactory (5)
Greversen et al. [46]	Two surgeons after 50 PIPAC procedures	✓ Cisplatinum	Pt/blood samples	Not provided	To assess Pt contamination in PIPAC exposed subjects	Blood samples showed no traces of Pt	Unsatisfactory (3)
Friese et al. [4]	One academic medical center/ambulatory oncology department/nurses, medical assistants, pharmacists, and PTs present during a drug spill (9) or working in a cancer center without experiencing spills (8)	✓ VP- 16 ✓ Docetaxel ✓ Pemetrexed	VP-16, Docetaxel and pemetrexed/urine/8 h urine samples collected when a spill of ADs occurred or in the period 4 h before and 4 h after the end of the shift	LC-MS/MS LOQ (ng/mL): VP-16 0.02; Docetaxel 0.025; Pemetrexed 0.109	Evaluate the internal dose of ADs after spills and in ordinary conditions of a cancer center activity	Workers with VP-16 exposure: 1/6 urine samples >LOD, but not the LOQ. No detectable levels in samples from workers without drug spill exposure. Workers with docetaxel, pemetrexed and cisplatin exposure: 3/3 samples from workers > LOD for docetaxel, no samples > LOD for pemetrexed. All these samples were >LOQ (drug levels: 0.58 and 0.10 ng/mL). Four samples from workers who did not report a drug spill were >LOD for docetaxel, but not >LOQ.	Satisfactory (6)
Hon et al. [47]	Five acute care sites and one cancer treatment centre of Canada/healthcare workers (103) as pharmacists, pharmacy receiver, PT, nurse, transport staff, unit clerks, and others working in drug administration units. Male/female: 21%/82%. Time period:June 2010–February 2011.	✓ CPA	Unmetabolized CPA/urine	UHPLC-MS/MS LOD (ng/mL):0.05	To quantify the urine concentration of non-metabolized CPA, among potentially exposed Canadian healthcare workers	111 of the 201 urine collected samples (55%) had levels greater than the LOD of 0.05 ng/mL. Maximum reported CPA concentration: 2.37 ng/mL; mean urinary CPA concentration: 0.156 ng/mL	Satisfactory (6)
Ramphal et al. [48]	A single pediatric hospital, Ottawa, Canada/personnel in the oncology pharmacy (7 who handled CPA on the day of the study participation), and non-oncology pharmacy personnel not exposed to CPA (5as controls).	✓ CPA	CPA/urine/24 h urine samples	GC/MS	To assess levels of CPA in exposed and not exposed pharmacy personnel	All participants in both groups tested positive for CPA, with a higher mean concentration in the urine of controls (mean range: 30–108.3 ng/mL) compared to the exposed personnel (mean range: 5–66.5 ng/mL).	Satisfactory (6)
Villa et al. [49]	Two hospitals performing HIPEC in France, Paris/exposed members of the surgical staff (senior and junior surgeons, anesthesiologist, operating room nurse, nurse anesthesist), the operating room cleaner and the staff member who transported drugs from the pharmacy to the opearting room (29 workers, 14 F and 15 M; aged 27–59 years). Healthcare workers (7 workers, 4 F and 3 M; aged 21–53 years) from the same hospitals were enrolled as unexposed controls.	✓ Oxaliplatinum	Pt/urine/ samples collected from the first void in the morning after the procedure	ICP-MS LOD (ng/mL):0.05 LOQ (ng/mL): 0.016	To assess levels of Pt in exposed and not exposed healthcare workers	Pt was undetectable (<0.05 ng/mL) in all workers. The Pt concentration was between the LOD and the LOQ in one of the 42 samples collected before HIPEC; the worker concerned had participated in another HIPEC procedure one month previously. In controls, Pt concentration was <LOD.	Satisfactory (6)
Sessink et al. [13]	A University Hospital in Brussels, Belgium/PTs (2) handling a robotic system for a part of the intravenous AD preparation. Time period:20 to 22 February 2022	✓ CPA	CPA/urine/24 h urine samples (10)	Analytical technique: not specified LOD (ng/mL):0.01	To assess levels of CPA in workers handling a robotic system for a part of the intravenous antineoplastic drug preparation	CPA was not detected in the 14 urine samples of the two technicians indicating no measurable exposure.	Unsatisfactory (4)

5-FU, 5-fluorouracil; AD, antineoplastic drug; CPA, cyclophosphamide; DNR, daunorubicin; DXR, doxorubicin; EPI, epirubicin; ESI-LC-MS, liquid chromatography electrospray ionization tandem mass spectrometric; ESI- UHPLC, negative electrospray ionization- ultra high performance liquid chromatography; FBAL, alpha-fluoro-beta-alanine; GCA, gemcitabine; GC-ECD, capillary gas chromatography with electron capture detection; GC-MS, gas chromatography-mass spectrometry; HIPEC, hyperthermic intraperitoneal chemotherapy; IP, ifosfamide; IRT, irinotecan; LA-ICP-MS, laser ablation inductively coupled plasma mass spectrometry; MRM-IDA-EPI, multiple reaction monitoring-information dependent acquisition enhanced production ion; MTX, methotrexate; PIPAC, Pressurized intraperitoneal aerosol chemotherapy; PT, pharmacy technicians; PTX, paclitaxel; RBC, red blood cell; UHPLC, ultra high performance liquid chromatography; VP-16, etoposide.

## Data Availability

Not applicable.

## Supplementary Materials

The following supporting information can be downloaded at: https://www.mdpi.com/article/10.3390/ijerph19063737/s1, Figure S1: Flow diagram of literature search.
