# Supplementary figures and images for: Exposure to Antineoplastic Drugs in Occupational Settings: A Systematic Review of Biological Monitoring Data
Source: Int J Environ Res Public Health. 2022 Mar 21;19(6):3737. doi: 10.3390/ijerph19063737 (PMC8952240; doi:10.3390/ijerph19063737)

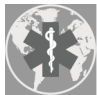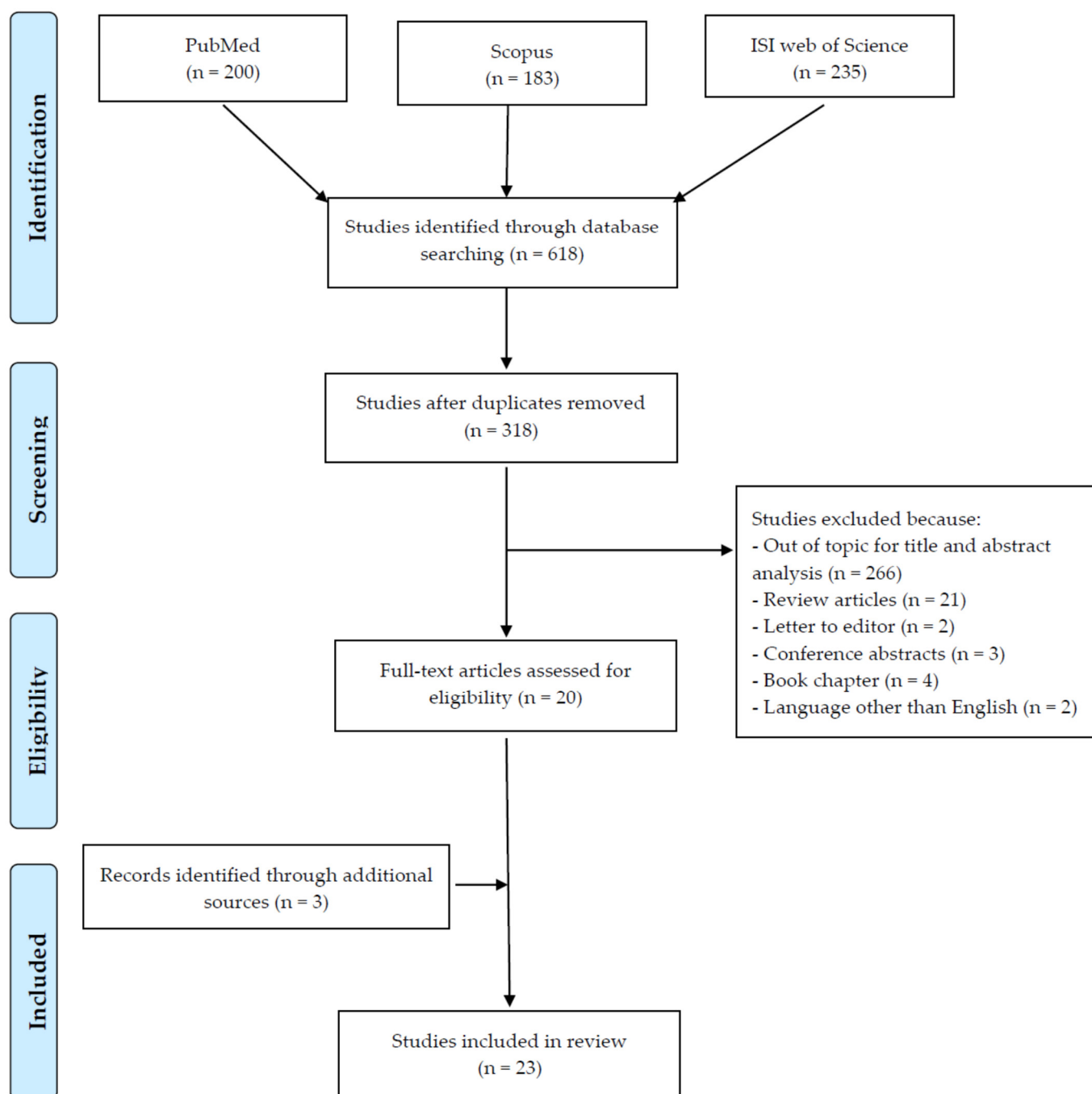

**Figure S1.** Flow diagram of literature search.

Supplement: Supplementary file 1 [file ijerph-19-03737-s001.zip › ijerph-1620832-supplementary.pdf]
